# Specifications of the ACMG/AMP Variant Curation Guidelines for Hereditary Hemorrhagic Telangiectasia Genes—*ENG* and *ACVRL1*

**DOI:** 10.1155/2024/3043736

**Published:** 2024-05-18

**Authors:** Desiree DeMille, Jamie McDonald, Carmelo Bernabeu, Hilary Racher, Carla Olivieri, Claudia Cantarini, Anna Sbalchiero, Bryony A. Thompson, Luca Jovine, Claire L. Shovlin, Sophie Dupuis-Girod, Gaetan Lesca, Maud Tusseau, Arupa Ganguly, Raj S. Kasthuri, Jaime Jessen, Maarten P. G. Massink, Shoji Ichikawa, Pinar Bayrak-Toydemir

**Affiliations:** ^1^Genomics Analysis, ARUP Laboratories, Salt Lake City, UT 84108, USA; ^2^Department of Pathology, University of Utah, Salt Lake City, UT 84108, USA; ^3^Centro de Investigaciones Biológicas Margarita Salas, Consejo Superior de Investigaciones Científicas (CSIC), 28040 Madrid, Spain; ^4^Impact Genetics/Dynacare, Brampton, Canada L6T 5M3; ^5^Department of Laboratory Medicine and Pathobiology, University of Toronto, Toronto, Canada M5S 1A8; ^6^Department of Molecular Medicine, University of Pavia, Pavia 27100, Italy; ^7^Department of Pathology, Royal Melbourne Hospital, Melbourne 3050, Australia; ^8^Department of Biosciences and Nutrition, Karolinska Institutet, Huddinge 141 83, Sweden; ^9^National Heart and Lung Institute, Imperial College, London W12 0HN, UK; ^10^Hospices Civils de Lyon, National HHT Reference Center and Department of Medical Genetics, Femme Mère Enfants Hospital, 69500 Bron, France; ^11^Department of Genetics, PSOM, University of Pennsylvania, Philadelphia, PA 19104, USA; ^12^Department of Medicine, University of North Carolina at Chapel Hill, Chapel Hill, NC 27599, USA; ^13^Department of Genetics, University Medical Center Utrecht, Utrecht 3584CX, Netherlands; ^14^Ambry Genetics, Aliso Viejo, CA 92656, USA

## Abstract

The 2015 ACMG/AMP standards and guidelines for interpretation of sequence variants are widely used by laboratories, including for variant curation of the hereditary hemorrhagic telangiectasia (HHT) genes. However, the need for gene- and disease-specific modifications and specifications of these general guidelines to optimize and standardize variant classification was recognized at the time of publication. With this goal, the ClinGen HHT variant curation expert panel was formed. Here, we describe our recommended HHT-specific variant classification criteria and the outcomes from pilot testing of 30 variants of the *ENG* and *ACVRL1* genes. Eight of the original ACMG/AMP rules were determined to not be applicable for *ENG*- or *ACVRL1*-related HHT or were previously recommended by ClinGen for removal, two rules were unmodified, and the remaining 18 rules were modified according to HHT specifications or previous ClinGen general recommendations. This study demonstrates the importance of HHT-specific criteria in the optimization and standardization of HHT variant classification and conflicting classification resolution.

## 1. Introduction

The 2015 standards and guidelines for interpretation of sequence variants published by the American College of Medical Genetics and Genomics (ACMG) and the Association for Molecular Pathology (AMP) are widely used by laboratories [1]. However, these general guidelines lack gene- and disease-specific specifications allowing for curator subjectivity leading to conflicting variant classifications. The anticipated need for gene- and disease-specific modifications and specifications of these general guidelines to optimize and standardize variant classification has been recognized by many genetic laboratories. With this goal, the NIH-Clinical Genome Resource (ClinGen) developed a process for convening teams of experts in different clinical domains referred to as variant curation expert panels (VCEPs). These expert panels are tasked with developing disease/gene-specific criteria for evaluating pathogenicity, as well as curation and review of variants in the ClinVar database—a public archive of reports of the relationships among human variations and phenotypes with supporting evidence. Using this framework, a panel of individuals with diverse areas of expertise related to hereditary hemorrhagic telangiectasia (HHT) was convened to provide HHT-specific rule modifications.

Pathogenic germline variants in *ACVRL1* and *ENG* cause HHT (MIM: 600376, 187300) and in *SMAD4* cause juvenile polyposis/HHT syndrome (JPS/HHT; MIM: 175050). HHT is an autosomal dominant disorder characterized by vascular malformations which lack normal capillary connections between artery and vein, referred to as telangiectases when small (typically in cutaneous or mucosal tissue), and arteriovenous malformations (AVMs) when larger (typically in solid organs). The prevalence is estimated to be 1/5,000 [2]. Penetrance is age dependent. As reported by those diagnosed with HHT, the penetrance of at least one manifestation approaches 100% by age 35 [3]. Clinical expression is highly variable, and many affected individuals remain undiagnosed [4]. Rare *de novo* [5, 6] and mosaic [7–11] cases have been reported.

Consensus clinical diagnostic criteria for HHT are referred to as the Curaçao Criteria [2, 12] and require at least 3 of the following:

1. Epistaxis: spontaneous and recurrent
2. Telangiectases: multiple at characteristic sites (lips, oral cavity, fingers, and nose)
3. Internal lesions: such as gastrointestinal telangiectasia; pulmonary, cerebral, hepatic, and spinal AVMs
4. Family history: first degree relative with HHT according to these criteria

The Curaçao Criteria lack sensitivity in childhood due to age-related penetrance of the most common symptoms of HHT [13–15]. In diagnosed individuals, epistaxis and telangiectases at these characteristic sites develop in more than 90% by age 40; yet only 50% of diagnosed individuals report having nosebleeds by age 10 [16, 17]. It has been shown, however, that in 91% of children proven by molecular diagnosis to have HHT, nasal telangiectases are seen using nasal endoscopy [18]. Oral/cutaneous telangiectases are often not noticed until the third decade of life [3, 19] and, even then, are usually appreciated only by focused examination. The number of telangiectases increase with age. In summary, HHT is underdiagnosed clinically, and a long diagnostic delay is common for those in whom the diagnosis is made [20, 21].

The phenocopy rate is low (0.15%) when the Curaçao Criteria are strictly applied and met. However, epistaxis and cutaneous red lesions are not uncommon in the general population and overlap in frequency (nosebleeds) and number (telangiectases) to that observed in HHT [17]. Thus, it is not uncommon to miscall individuals in a family already known to have HHT as “affected,” based solely on report of cutaneous telangiectases and/or epistaxis [22]. Furthermore, HHT overlaps clinically with capillary malformation-arteriovenous malformation (CM-AVM), including *RASA1*-related CM-AVM and more particularly *EPHB4*-related CM-AVM. Although epistaxis and cutaneous telangiectases are also features of CM-AVM, the cutaneous lesions have distinct features relative to HHT. A typical older adult with HHT has 1-2 dozen pinpoint to pinhead sized pink-red lesions in particular locations (fingers, lips, and oral cavity). In contrast, telangiectases “too numerous to count,” haloed, on the limbs and/or trunk, or with significant pediatric onset are not characteristic of HHT and are suggestive in particular of *EPHB4*-related CM-AVM [23]. Additionally, a partial phenotypic overlap with *GDF2*-related vascular anomaly syndrome is reported [24].

One study of consecutive cases identified at an HHT Center of Excellence as “definite” HHT according to strictly applied Curaçao Criteria found a causative variant in *ACVRL1* or *ENG* in ~96% and in *SMAD4* in ~1% of cases [25]. Multiple series that included cases of “suspected” as well as “definite” HHT, or in which clinical diagnostic criteria were applied by clinicians with a wide range of experience regarding HHT, have yielded a causative variant detection rate for these three genes of 75-85% [26–29]. Missense variants, nonsense variants, small insertions/deletions, single-nucleotide variants leading to splicing defects, large deletions/duplications, and chromosomal rearrangements are all described in *ACVRL1*, *ENG*, and *SMAD4*. Additional pathogenic regions outside of the intron/exon boundaries that should be interrogated during genetic analysis include *ACVRL1* intron 9 encompassing the CT-rich variant hotspot region near the exon 10 acceptor site [30] and *ENG* 5′ untranslated region (c.-10C>T, c.-127C>T, and other variants that create new alternative ATG codons predicted to affect translation initiation) [31–33].

This study focuses on the *ACVRL1* and *ENG* genes and does not include rule specifications for *SMAD4* or the other genes related to disorders with phenotypic overlap. Given the overlap of JP/HHT syndrome and juvenile polyposis (JP) syndrome, our group is collaborating with the ClinGen InSiGHT Hereditary Colorectal Cancer/Polyposis expert panel to provide *SMAD4* rule modifications.

## 2. Materials and Methods

The ClinGen HHT VCEP membership is composed of individuals with a wide range of HHT-specific expertise and includes clinical variant scientists, molecular geneticists, medical geneticists, disease specialists, genetic counselors, structural biologists, and basic science researchers. All expert panel members disclosed potential conflicts of interest as required by ClinGen. Expert members held discussions over email, teleconference, and in-person meetings to discuss rule modifications in context of HHT disease. Modifications and specifications were decided based on group consensus. Preliminary rules were piloted and adjusted as needed prior to submission for final approval by the ClinGen Sequence Variant Interpretation (SVI) Working Group. All reported variants are based off the following transcripts: *ACVRL1* (NM_000020.3) and *ENG* (NM_001114753.3).

## 3. Results and Discussion

### 3.1. HHT-Specific Variant Curation Rules

The HHT VCEP final rule specifications approved by the ClinGen SVI Working Group are summarized in Table 1. Eight rules were determined to not be applicable for *ENG*- or *ACVRL*1-related HHT or were previously recommended for removal by the ClinGen SVI Working Group (e.g., PP5 and BP6) [34]. Two rules were unmodified and can be used as originally described in Richards et al. [1] (PS1, PM4). The remaining 18 rules were modified either according to HHT specifications, or previous ClinGen SVI general recommendations (e.g., PM2 modified to PM2_Supporting; see https://clinicalgenome.org/working-groups/sequence-variant-interpretation/). Two new rules for combining criteria codes were also added to the original 2015 guidelines (Table 2). This is to account for the decrease in PM2 weight from moderate evidence to supporting and to be able to classify variants as likely benign if they meet BS1 level evidence.

### 3.2. Evidence Assessment

#### 3.2.1. Null Variants (PVS1)

**Figure 1:**
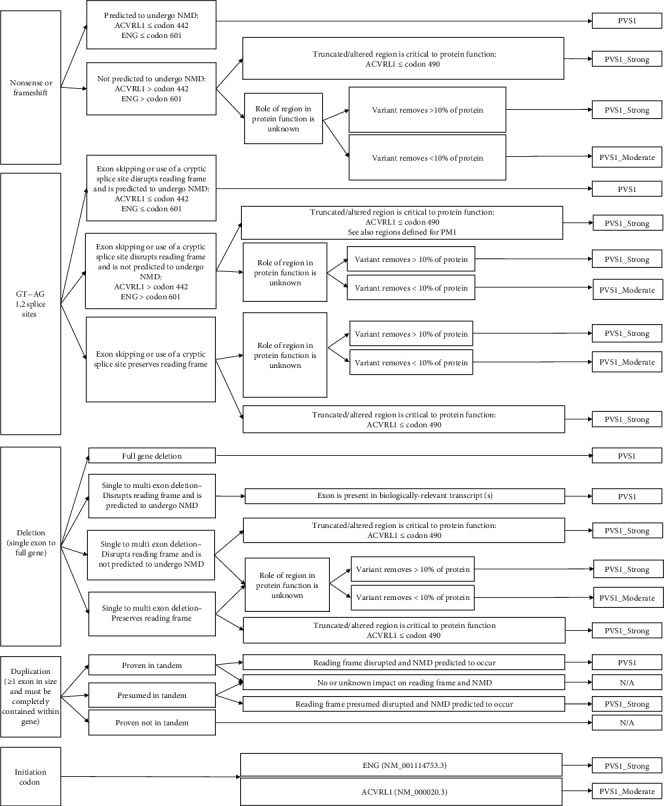
HHT PVS1 decision tree adapted from Abou Tayoun et al. [35].

According to Abou Tayoun et al. [35], initiation codon variants are not recommended to reach higher than PVS1_Moderate level evidence. Variants in the initiation codon or in the noncoding exon 1 of *ACVRL1* (NM_000020.3), to date, have not been reported in association with HHT. Since rescue resulting from the next in-frame ATG at codon 12 is a possibility and the function of codons 1-11 of ACVRL1 are unknown, the highest strength level of an initiation codon variant seen in *ACVRL1* is recommended to be PVS1_Moderate (refer to Figure 1). In contrast, initiation codon variants in *ENG* (e.g., c.1A>G, c.2T>G, and c.2T>C) have been reported in several individuals affected with HHT and are considered causative. Thus, we recommend applying PVS1_Strong evidence for initiation codon variants in *ENG* (NM_001114753.3) (refer to Figure 1).

#### 3.2.2. Population Data (BA1, BS1, and PM2)

HHT is not known to be enriched in bottlenecked populations (e.g., Ashkenazi Jewish); therefore, Popmax/Grpmax filtering allele frequency (FAF) can be calculated and applied for bottlenecked populations for BA1, BS1, and BS1_Supporting criteria. The HHT ClinGen VCEP was conservative in setting the population frequency thresholds required as evidence that a variant is benign given that the 1/5,000 prevalence estimate of HHT may be an underestimate due to underdiagnosis. Additionally, the *ENG* c.-9G>A variant has been reported in affected individuals and may affect translation efficiency [25, 28, 31]. This variant is found in the Genome Aggregation Database (gnomAD v2.1.1) at a frequency of 0.08% (76/95698 alleles) in the European population. Given that this variant may cause HHT in some individuals and that there may be additional mild HHT variants, the population thresholds were set accordingly (refer to Table 1).

HHT exhibits age-related penetrance with a clinical variability which includes mildly affected, undiagnosed individuals even in adulthood. A few reported pathogenic HHT variants have 4-5 alleles reported in gnomAD v2.1.1. Thus, PM2_Supporting criteria may be applied if the variant has <6 total alleles or is <0.00004 (0.004%) in a gnomAD (v2.1.1) subpopulation (containing >1,000 individuals).

#### 3.2.3. *De Novo* (PS2)

*(1) PS2*. It may be applied if the variant is *de novo* (both maternity and paternity confirmed) in a patient with the disease and no family history. Due to the highly variable phenotype and age-related penetrance in both *ENG*- and *ACVRL1*-related HHT, to be considered unaffected in applying *de novo* evidence, the parents should be over age 40 and have no history of recurrent epistaxis or telangiectases based on targeted questioning and physical examination. Caution must also be used as low-level mosaicism has been observed in parents of individuals with HHT which may not be detectable by Sanger sequencing or NGS [7–10].

#### 3.2.4. Proband Counting (PS4, PP4)

*(1) PS4*. The proband counting criterium (PS4, PS4_Moderate, and PS4_Supporting) may be applied if there are one or more probands with a phenotype consistent with HHT (refer to Table 1). In general, individuals should be reported to have at least two manifestations to be included for the purpose of applying this criterium. Reported history of nosebleeds alone should not be considered sufficient in application of this rule since 11% of the general population reports six or more nosebleeds per year [36]. In contrast, manifestations with low phenocopy rates (e.g., pulmonary AVMs [4] or chronic severe nose bleeding requiring intervention) would be considered particularly suggestive of the diagnosis. Precapillary pulmonary arterial hypertension (PAH) is rarely associated with HHT, particularly but not exclusively in association with pathogenic variants in the *ACVRL1* gene [37, 38].

*(2) PP4_Moderate*. Application of this rule requires that the patient's phenotype meets consensus clinical diagnostic (Curaçao) Criteria for HHT (see HHT introduction section) and that sequencing and large deletion/duplication analysis was performed for both *ENG* and *ACVRL1* with no other causative variant identified. *ACVRL1* and *ENG* are each responsible for approximately half of confirmed HHT cases and a majority of cases combined [25–29]; therefore, both need to be tested to rule out a causative variant in the other gene. *SMAD4* is responsible for a small percentage of HHT cases (1-2%) and application of PP4_Moderate rule does not require its analysis.

Note: PP4_Moderate cannot be applied to variants that meet BA1, BS1, or BS1_Supporting criteria. If PP4_Moderate can be applied to a patient, they cannot be included in proband counting (PS4).

#### 3.2.5. Functional Evidence (PS3, BS3)

All currently known HHT-causative genes code for members of the transforming growth factor-*β* (TGF-*β*) signaling pathway [39, 40]. Endoglin (*ENG*) and ALK1 (*ACVRL1*) form a receptor complex expressed on the surface of endothelial cells where they bind circulating BMP9 (*GDF2*) and BMP10 ligands, the BMP9/BMP10 heterodimers accounting for most of their signaling activity in plasma [41]. Upon ligand binding, the kinase ALK1 is activated to phosphorylate the transcription factors SMAD1/5. In turn, phospho-SMAD1/5 associates with SMAD4, and the resulting complex translocates to the nucleus to regulate the expression of multiple downstream target genes [42].

There are a limited number of either *ENG*- or *ACVRL1*-related HHT variants classified pathogenic or benign for which there is functional data. This limitation makes it difficult to determine a false positive/false negative/true positive/true negative rate to determine the reliability of previously performed assays. With the data that is available, functional results have largely matched variant classifications inferred from other criteria (phenotype, cosegregation, etc.) for the following assays listed below. PS3, PS3_Supporting, and BS3_Supporting criteria may be applied for the following:

(i) PS3: mRNA splicing assays can be used as strong functional evidence. Note: level of evidence used may differ depending on whether the abnormal transcript is in-frame or out-of-frame and whether there is complete or incomplete splicing impact
  - Note: do not use PS3 for canonical splice variants (+/-1,2) that meet PVS1
(ii) PS3_Supporting: all other previously performed *ENG*- or *ACVRL1*-related HHT assays can be used as supporting evidence and increased to moderate/strong criteria if multiple different functional assays are concordant
  (a) Protein expression assays: metabolic label (ML) and immunoprecipitation (IP); western blot (WB) and fluorescence-activated cell sorting (FACS) of human umbilical cord endothelial cells (HUVECs)/blood outgrowth endothelial cells (BOECs); FACS of activated monocytes; cDNA transfect, WB and ML HEK293T/COS/NIH3T3; cDNA transfect and luciferase assay in HepG2 cells
    1. Note: decreased protein expression can be used as supporting pathogenic evidence if an experiment was not done in a single assay, and the corresponding densitometry of western blot reflects the conclusion drawn
  (b) Intracellular signaling assays: BRE/CAGA-luciferase and Gal4 Smad1/Smad3 for TGF-*β*/BMP9 signaling
  (c) Binding assays: BMP9 binding, transcription factor Sp1, and BMP9 protein-protein interaction by biolayer interferometry (BLI)
  (d) Subcellular protein localization
  (e) Morphology: cell morphology, actin cytoskeleton organization, and tubulogenesis
(iii) BS3_Supporting: all previously performed *ENG*- or *ACVRL1*-related HHT assays can be used as supporting evidence
  - mRNA splicing assays
  - Intracellular signaling assays: BRE/CAGA-luciferase and Gal4 Smad1/Smad3 for TGF-*β*/BMP9 signaling
  - Binding assays: BMP9 binding, transcription factor Sp1, and BMP9 protein-protein interaction (BLI)
  - Subcellular protein localization
  - Morphology: cell morphology, actin cytoskeleton organization, and tubulogenesis

Note: normal protein expression cannot be used as benign evidence because protein function can still be altered (e.g., pathogenic dominant negative variants).

#### 3.2.6. Functional Domains/Regions (PM1)

*(1) PM1*. This evidence may be applied if the variant falls within a critical residue listed for each gene below. Each of these regions lacks high population frequency missense variants in gnomAD v2.1.1, has likely pathogenic or pathogenic variants reported in the region, and has sequence homology and/or structural analysis thought to be critical for protein folding and/or function [43–47].

(i) *ACVRL1* (ALK1)
  (a) BMP9/10 interaction site residues:
    1. His40, Val54, Val56, Arg57, Glu58, Glu59, His66, Asn71, Leu72, His73, Glu75, Leu76, Arg78, Gly79, Arg80, Thr82, Glu83, Phe84, Val85, and His87
  (b) Glycine-rich loop: Gly209-Val216
  (c) Phosphate anchor: Lys229
  (d) C-helix E pairing the phosphate anchor: Glu242
  (e) Catalytic loop: Arg329-Asn335
  (f) Metal-binding loop: Asp348-Leu351
(ii) *ENG* (endoglin)
  (a) BMP9 binding site residues: Ser278 and Phe282
  (b) Cysteine residues previously reported to be likely pathogenic or pathogenic:
    - Cys207, Cys363, Cys382, Cys412, and Cys549
  (c) Cysteine residues known to be important for ENG folding:
    - Cys350 (Cys350-Cys382 disulfide in ZP-N domain of ENG is required for secretion of its ZP module)
    - Cys394 (makes a disulfide bond with Cys412 which is reported to be a mutated residue)
  (d) Cysteine residues known to be important for ENG function:
    - Cys516 (involved in forming intermolecular disulfides that hold ENG homodimer together)
    - Cys582 (involved in forming intermolecular disulfides that hold ENG homodimer together)

Note: if the variant falls within a PM1 region, do not use PM1 with PM5_Strong. PM1 can still be combined with PM5.

#### 3.2.7. Segregation (PP1, BS4)

If the variant under assessment segregates with an HHT phenotype in one or more families, PP1 may be applied as follows:

- PP1_Strong: 5 meioses (1/32 likelihood)
- PP1_Moderate: 4 meioses (1/16 likelihood)
- PP1: 3 meioses (1/8 likelihood)

It is important to note that assignment of affected or unaffected status to family members must consider that the most common manifestations of HHT (nosebleeds/epistaxis and telangiectases) have significant phenocopy rates, age-related penetrance, and highly variable expression.

The occurrence of epistaxis and telangiectases in the general population (phenocopies), and mosaicism in clinically affected individuals, can mimic lack of segregation among affected individuals. Thus, the ClinGen HHT VCEP recommends the following for use of affected/unaffected status for purpose of inclusion in a cosegregation study:

Affected: 3 or more manifestations of HHT (first-degree relative with HHT by Curaçao Criteria, counts as one).

Unaffected: do not include for the purpose of segregation analysis. On clinical grounds, an individual cannot at any age be assigned unaffected status with confidence [4, 21].

#### 3.2.8. Alternate Molecular Cause (BP2, BP5)

*(1) BP2*. It may be applied if the variant under assessment is observed *in trans* with a likely pathogenic or pathogenic variant based on HHT VCEP rules. Do not apply if the variant is observed in cis since its effect would be unknown, if found alone.

*(2) BP5*. It may be applied if the variant under assessment is found with a likely pathogenic or pathogenic variant (based on HHT VCEP rules) in a different gene, and the different gene is either *ACVRL1* or *ENG*.

#### 3.2.9. Computational (BP4, BP7)

BP4 and BP7 can be combined for synonymous or intronic variants where splicing prediction algorithms predict no impact to the splice consensus sequence nor the creation of a new splice site. However, our group cautions that if no causative variant is found and the patient's clinical presentation and/or family history is highly suspicious for HHT, be careful not to dismiss intronic variants or synonymous variants in the last nucleotide of the exon based on computational predictions. In example 1, *ENG* c.219G>A; p.Thr73= is not predicted to significantly alter splicing (SpliceAI: 0.02) and the nucleotide is weakly conserved. However, this variant was later shown to cause exon skipping ([48], ARUP Laboratories). In example 2, SpliceAI does not predict splicing effects for some deep intronic *ACVRL1* intron 9 CT rich hotspot variants [30]. Therefore, variants that create a new “AG” cryptic splice site in this region should not be ruled out based on SpliceAI prediction alone.

### 3.3. Rule Piloting

Thirty *ENG* and *ACVRL1* sequence variants were selected for the piloting of our modified rules. Variants were selected to include well-established pathogenic and benign variants, variants with conflicting classifications in the ClinVar database, and different types of variants (missense, intronic, UTR, nonsense, frameshift, and initiation codon). At least three independent biocurators performed curation for each variant. If the final classifications and evidence codes used were concordant, the variant was not further discussed. If results between the three biocurators were conflicting, additional biocurators and group discussions were utilized until a majority opinion was reached. A summary of piloted variants, ClinVar and HHT VCEP classifications, and evidence codes applied are shown in Table 3.

## 4. Conclusions

The work of the ClinGen HHT VCEP presented here aids in the standardization of variant classification and data sharing of HHT variants to the ClinVar database. Future work of the HHT VCEP is to continue classifying variants within the ClinVar database using *ENG*- and *ACVRL1*-related HHT-specific rule modifications to help provide a central, curated resource where clinicians and researchers can go to find the significance of variants associated with *ENG*- and *ACVRL1*-related HHT. The rule modifications presented herein are considered a first version and will be published in the Criteria Specification (CSPEC) registry which can be accessed from the ClinGen HHT VCEP page (https://clinicalgenome.org/affiliation/50037/). As updates to the general ACMG/AMP variant classification guidelines are published, the ClinGen HHT VCEP will continue to refine and improve the *ENG*- and *ACVRL1*-related HHT-specific variant classification guidelines and future versions will be available on the CSPEC registry.

## Figures and Tables

**Table 1:** Summary of gene-specific criteria for *ACVRL1* and *ENG* variant classification.

Criteria code	Original ACMG summary	HHT modifications
*Pathogenic criteria*		
PVS1	Null variant (nonsense, frameshift, canonical +/−1 or 2 splice sites, initiation codon, single or multiexon deletion) in a gene where loss of function (LOF) is a known mechanism of disease.	Use decision tree adapted from Abou Tayoun et al. [35] (Figure 1).
PS1	Same amino acid change as a previously established pathogenic variant regardless of nucleotide change.	No modification.
PS2	De novo (both maternity and paternity confirmed) in a patient with the disease and no family history.	See HHT screening requirements to be met prior to considering parents unaffected in Evidence Assessment.
PS3	Well-established in vitro or in vivo functional studies supportive of a damaging effect on the gene or gene product.	PS3: mRNA splicing assays. PS3_Supporting: all other functional assays. Note: evidence strength may be increased to moderate/strong if multiple different functional assays are concordant. See Evidence Assessment for details.
PS4	The prevalence of the variant in affected individuals is significantly increased compared to the prevalence in controls.	PS4: 4+ probands with phenotype consistent with HHT. PS4_Moderate: 2-3 probands with phenotype consistent with HHT. PS4_Supporting: 1 proband with phenotype consistent with HHT. See Evidence Assessment for HHT phenotype requirements.
PM1	Located in a mutational hot spot and/or critical and well-established functional domain (e.g., active site of an enzyme) without benign variation.	See Evidence Assessment for critical regions and details.
PM2	Absent from controls (or at extremely low frequency if recessive) in Exome Sequencing Project, 1000 Genomes, or Exome Aggregation Consortium.	PM2: modified to supporting per ClinGen SVI recommendation. PM2_Supporting: if the variant has <6 total alleles or has an allele frequency of <0.00004 (0.004%) in gnomAD (v2.1.1) subpopulations (containing >1,000 individuals), this evidence can still be applied. See additional details in Evidence Assessment.
*PM3*	*For recessive disorders, detected in trans with a pathogenic variant.*	Removed: Not applicable; ENG- and ACVRL1-related HHT are autosomal dominant disorders.
PM4	Protein length changes due to in-frame deletions/insertions in a nonrepeat region or stop-loss variants.	No modification.
PM5	Novel missense change at an amino acid residue where a different missense change determined to be pathogenic has been seen before.	PM5_Strong: ≥2 different missense changes determined to be likely pathogenic or pathogenic based on HHT VCEP rules. PM5: a different missense change determined to be likely pathogenic or pathogenic based on HHT VCEP rules. Note: if the variant falls within a PM1 region, do not use PM1 with PM5_Strong.
*PM6*	*Assumed de novo, but without confirmation of paternity and maternity.*	Removed: Not applicable; de novo variants are rare in ENG- and ACVRL1-related HHT. De novo variants should be confirmed not assumed for HHT.
PP1	Cosegregation with disease in multiple affected family members in a gene definitively known to cause the disease.	PP1_Strong: 5 meioses (1/32 likelihood). PP1_Moderate: 4 meioses (1/16 likelihood). PP1: 3 meioses (1/8 likelihood). See requirements for cosegregation studies in Evidence Assessment.
*PP2*	*Missense variant in a gene that has a low rate of benign missense variation and where missense variants are a common mechanism of disease.*	Removed: Does not apply to ENG (gnomAD v2.1.1 Z-score 0.93) or ACVRL1 (gnomAD v2.1.1 Z-score 2.45).
PP3	Multiple lines of computational evidence support a deleterious effect on the gene or gene product (conservation, evolutionary, splicing impact, etc.).	For missense variants: REVEL score ≥0.644 or SpliceAI ≥0.2. For synonymous and intronic variants: SpliceAI ≥0.2.
PP4	Patient's phenotype or family history is highly specific for a disease with a single genetic etiology.	PP4_Moderate: patient's phenotype meets Curaçao Criteria for HHT, and sequencing and large deletion/duplication analysis was performed for ENG and ACVRL1. See Evidence Assessment for additional details and requirements.
*PP5*	*Reputable database reports variant as pathogenic but without evidence to independently evaluate.*	Removed: SVI Working Group recommendation [34].

*Benign criteria*		
BA1	Allele frequency is above 5% in Exome Sequencing Project, 1000 Genomes, or Exome Aggregation Consortium.	≥1% in general population databases (e.g., gnomAD (v2.1.1)) based on Popmax/Grpmax filtering allele frequency (FAF). See additional details in Evidence Assessment.
BS1	Allele frequency is greater than expected for disorder.	BS1: >0.2% to <1% in general population databases (e.g., gnomAD v2.1.1) based on Popmax/Grpmax FAF, OR if variant meets BS1_Supporting and has ≥2 homozygotes. BS1_Supporting: >0.08% to 0.2% (based on gnomAD (v2.1.1) Popmax/Grpmax FAF). See additional details in Evidence Assessment.
*BS2*	*Observed in a healthy adult individual for a recessive (homozygous), dominant (heterozygous), or X-linked (hemizygous) disorder, with full penetrance expected at an early age.*	Removed: Not applicable; full penetrance at an early age is not typical in HHT.
BS3	Well-established in vitro or in vivo functional studies show no damaging effect on protein function or splicing.	BS3_Supporting: all functional assays. See Evidence Assessment for details.
BS4	Lack of segregation in affected members of a family.	See requirements described in Evidence Assessment.
*BP1*	*Missense variant in a gene for which primarily truncating variants are known to cause disease.*	Removed: Not applicable; missense variants are commonly seen in the ENG and ACVRL1 genes.
BP2	Observed in trans with a pathogenic variant for a fully penetrant dominant gene/disorder or observed in cis with a pathogenic variant in any inheritance pattern.	Observed in trans with a likely pathogenic or pathogenic variant based on HHT VCEP rules. See Evidence Assessment for details.
*BP3*	*In-frame deletions/insertions in a repetitive region without a known function.*	Removed: Not applicable.
BP4	Multiple lines of computational evidence suggest no impact on gene or gene product (conservation, evolutionary, splicing impact, etc.).	For missense variants: REVEL score ≤0.15 and SpliceAI ≤0.1. For synonymous and intronic variants: SpliceAI ≤0.1. See Evidence Assessment for details.
BP5	Variant found in a case with an alternate molecular basis for disease.	Observed in a case with a likely pathogenic or pathogenic variant (based on HHT VCEP rules) in a different gene and the different gene is either ACVRL1 or ENG. See Evidence Assessment for details.
*BP6*	*Reputable database reports variant as benign but without evidence to independently evaluate.*	Removed: SVI Working Group recommendation [34].
BP7	A synonymous variant for which splicing prediction algorithms predict no impact to the splice consensus sequence nor the creation of a new splice site AND the nucleotide is not highly conserved.	For synonymous and intronic variants: SpliceAI ≤0.1. Can be used together with BP4 evidence. See Evidence Assessment for details.

**Table 2:** Rules for combining criteria. Rules marked with asterisk (^∗^) indicate addition from the original ACMG/AMP 2015 rule combinations.

Rules for combining pathogenic criteria
*Pathogenic*
(1) 1 very strong *AND*
(a) ≥1 strong *OR*
(b) ≥2 moderate *OR*
(c) 1 moderate *AND* 1 supporting *OR*
(d) ≥2 supporting
(2) ≥2 strong *OR*
(3) 1 strong *AND*
(a) ≥3 moderate *OR*
(b) 2 moderate *AND* ≥2 supporting *OR*
(c) 1 moderate *AND* ≥4 supporting
*Likely pathogenic*
(1) 1 very strong *AND*
(a) 1 moderate *OR*
(b) **1 supporting**^∗^***OR***
(2) 1 strong *AND*
(a) 1-2 moderate *OR*
(b) ≥2 supporting
(3) ≥3 moderate *OR*
(4) 2 moderate *AND* ≥2 supporting *OR*
(5) 1 moderate *AND* ≥4 supporting

Rules for combining benign criteria
*Benign*
(1) 1 stand‐alone *OR*
(2) ≥2 strong *OR*
*Likely benign*
(1) **1 strong (BS1)**^∗^***OR***
(2) 1 strong *AND* 1 supporting *OR*
(3) ≥2 supporting

**Table 3:** Summary of *ACVRL1* and *ENG* variant classifications from rule piloting.

Variant	ClinVar ID	ClinVar classifications^∗^ (# of submissions)	HHT VCEP classifications^∗^	Codes applied by HHT VCEP
*ACVRL1*				
c.88C>T (p.Pro30Ser)	161202	B (1), LB (4)	LB	BP2, BP5
c.113G>A (p.Ser38Asn)	1948619	VUS (1)	VUS	PM2_Supporting
c.137G>C (p.Cys46Ser)	533345	VUS (1), LP (1)	LP	PM2_Supporting, PP3, PS4
c.500C>G (p.Ser167Cys)	1744752	VUS (1), LP (1)	VUS	PM2_Supporting, PS4_Supporting, PP3
c.652C>T (p.Arg218Trp)	802861	B (3), LB (1)	LB	BS1, PP3
c.706G>A (p.Glu236Lys)	657805	VUS (1), LP (2), P (1)	P	PM2_Supporting, PS4, PP1_Strong, PP3
c.917C>T (p.Ala306Val)	811065	B (1), VUS (5)	VUS	None
c.982C>T (p.His328Tyr)	848699	LP (1), P (2)	LP	PM2_Supporting, PP3, PP4_Moderate, PS4_Moderate
c.998G>T (p.Ser333Ile)	212802	P (5)	P	PM1, PM2_Supporting, PS4, PS3_Supporting, PP1_Strong, PP3, PP4_Moderate
c.1217G>A (p.Trp406Ter)	411300	P (1)	P	PVS1, PM2_Supporting, PS4_Supporting
c.1232G>A (p.Arg411Gln)	8243	LP (1), P (11)	P	PS4, PM2_Supporting, PP3, PP1_Strong, PP4_Moderate, PS3_Supporting
c.1348A>G (p.Thr450Ala)	373609	B (1), LB (2), VUS (1)	LB	BS1
c.1377+4A>T	994236	VUS (1)	VUS	PM2_Supporting, PS4_Supporting, PP3
c.1445C>T (p.Ala482Val)	161201	B (5), LB (8), VUS (2)	LB	BS1, BP5, BS3_Supporting, PP3, PM5
c.1468C>T (p.Gln490Ter)	426040	P (3)	P	PVS1_Strong, PM2_Supporting, PP1_Strong, PS4

*ENG*				
c.-9G>A	414302	LB (2), VUS (4), LP (1), P (1)	VUS	PS3_Supporting, BP5
c.2T>G (p.?)	458346	P (2)	P	PM2_Supporting, PVS1_Strong, PS4, PP4_Moderate, PM5_Strong
c.1160T>C (p.Leu387Pro)	565574	VUS (1)	VUS	PM2_Supporting, PP3
c.1312-3C>G	1352569	VUS (1)	VUS	PM2_Supporting, PP3
c.1316A>C (p.Lys439Thr)	365088	B (1), VUS (2)	LB	BS1_Supporting, BP4, BS3_Supporting
c.1510G>A (p.Val504Met)	161231	B (6), LB (2), VUS (1)	LB	BS1, BP2, BS3_Supporting
c.1701del (p.Val568SerfsTer5)	618625	P (1)	P	PVS1, PM2_Supporting, PS4_Supporting
c.1711C>T (p.Arg571Cys)	282707	LB (1), VUS (3)	VUS	None
c.1844C>T (p.Ser615Leu)	161229	B (3), LB (11)	LB	BS1, BP5, BS3_Supporting
c.1961C>G (p.Thr654Ser)	426118	VUS (1)	VUS	PM2_Supporting, BP4
c.392C>T (p.Pro131Leu)	161232	B (7), LB (2), VUS (1)	B	BA1
c.447G>C (p.Trp149Cys)	237027	P (8)	P	PM2_Supporting, PP3, PP4_Moderate, PS4, PS3_Supporting, PP1
c.572G>A (p.Gly191Asp)	213200	B (10), LB (5)	B	BA1, BP5
c.662 T>C (p.Leu221Pro)	435060	LP (1), P (5)	LP	PM2_Supporting, PS4, PP4_Moderate, PS3_Supporting
c.991G>A (p.Gly331Ser)	407115	LP (2), P (8)	P	PM2_Supporting, PP1, PP4_Moderate, PS3, PS4

*ACVRL1* (NM_000020.3); *ENG* (NM_001114753.3). B: benign; LB: likely benign; VUS: variant of uncertain significance; LP: likely pathogenic; P: pathogenic. ^∗^Classifications as of December 21, 2023.

## Data Availability

ClinGen HHT VCEP rule modifications and future updates will be published in the Criteria Specification (CSPEC) registry and available to access from the ClinGen HHT VCEP page (https://clinicalgenome.org/affiliation/50037/).
